# 22(R)-hydroxycholesterol for dopaminergic neuronal specification of MSCs and amelioration of Parkinsonian symptoms in rats

**DOI:** 10.1038/s41420-020-00351-6

**Published:** 2021-01-16

**Authors:** Manisha Singh, Manish Jain, Samrat Bose, Ashutosh Halder, Tapas Chandra Nag, Amit Kumar Dinda, Sujata Mohanty

**Affiliations:** 1 Stem Cell Facility (DBT-Centre of Excellence for Stem Cell Research), All India Institute of Medical Sciences, New Delhi, 110029 India; 2 The Solomon H. Snyder Department of Neuroscience, Johns Hopkins University School of Medicine, Baltimore, MD USA; 3 Department of Reproductive Biology, All India Institute of Medical Sciences, New Delhi, 110029 India; 4 Department of Physiology, All India Institute of Medical Sciences, New Delhi, 110029 India; 5 Sophisticated Analytical Instrumentation Facility, All India Institute of Medical Sciences, New Delhi, 110029 India; 6 Department of Pathology, All India Institute of Medical Sciences, New Delhi, 110029 India

**Keywords:** Mesenchymal stem cells, Stem-cell research

## Abstract

Oxysterols play vital roles in the human body, ranging from cell cycle regulation and progression to dopaminergic neurogenesis. While naïve human mesenchymal stem cells (hMSCs) have been explored to have neurogenic effect, there is still a grey area to explore their regenerative potential after in vitro differentiation. Hence, in the current study, we have investigated the neurogenic effect of 22(R)-hydroxycholesterol (22-HC) on hMSCs obtained from bone marrow, adipose tissue and dental pulp. Morphological and morphometric analysis revealed physical differentiation of stem cells into neuronal cells. Detailed characterization of differentiated cells affirmed generation of neuronal cells in culture. The percentage of generation of non-DA cells in the culture confirmed selective neurogenic potential of 22-HC. We substantiated the efficacy of these cells in neuro-regeneration by transplanting them into Parkinson’s disease Wistar rat model. MSCs from dental pulp had maximal regenerative effect (with 80.20 ± 1.5% in vitro differentiation efficiency) upon transplantation, as shown by various behavioural examinations and immunohistochemical tests. Subsequential analysis revealed that 22-HC yields a higher percentage of functional DA neurons and has differential effect on various tissue-specific primary human MSCs. 22-HC may be used for treating Parkinson’s disease in future with stem cells.

## Introduction

Cholesterol is a type of sterol, which is biosynthesized in all types of animal cells and is an important part of the cell membrane. Human brain consists of as high as ~25% of the body’s total cholesterol^1^. Cholesterols self-assemble and form various structures, contributing to the varied functions of the brain^2^. The lipidome profile of central nervous system (CNS) is associated with neuronal activity, cognitive behaviour and various neurological disorders^3^. While cholesterol is required for dopaminergic (DAergic) neuronal maturity in terms of maintaining synapses and neurotransmission, they are also required for their survival^4,5^.

Oxysterols are the oxidized derivatives of cholesterols which show their effect through lxr-α and lxr-β. Lxr (liver X receptors) are nuclear receptors which function through their oxysterol ligands and control activities like cell division, ventral midbrain neurogenesis and DAergic neurogenesis^5^. The study reported by Paolla Sacchetti et al.^5^ states the presence of oxysterols like 24-HC and 25-HC in DAergic neurogenesis in mice brain. The same study reports the relevance of 22-HC in DAergic neurogenesis in human embryonic stem cell (hESC) lines, H9 and HS181. Oxysterols have also been reported to maintain the balance between neuronal and glial cells generation.

However, to date there is no report that states the effect of oxysterols in generation of DAergic neuronal cells from mesenchymal stem cells (MSCs). Furthermore, there are very few studies reporting the momentousness of transplantation of human mesenchymal stem cells (hMSCs) in Parkinson’s disease (PD) rat models. Most of the reported studies target the transplantation of undifferentiated MSCs in PD models of rat, mice or macaque^6^. While there are different tissue sources to obtain MSCs for transplantation^7–9^, there are varied reports on the inducers used to coax stem cells in culture^7,10–15^, mode of transplantation and duration of study^15^. To date, a number of PD animal models have been established to study the pathogenesis of the disease and test the possible drug or cell-based therapeutics^16^. There are two types of PD models being developed by using neurotoxins: (a) reversible PD model^6^ and (b) irreversible PD model. However, most of the recent studies focus on the use of irreversible PD models to study PD and therapeutics associated with it. Therefore, in this study, 6-OHDA will be used to create lesions in the midbrain of the Wistar rats. These neurotoxins hold the ability to produce an oxidative stress and cause cell death in DA neuronal population, which further, represents the symptoms of PD^17^.

Despite the fact that MSCs share similar characteristics, there are subtle variations due to which they show differential effect in different diseases. Thus, in this study the optimum effect of 22-HC on hMSCs obtained from various tissues has been identified. MSCs provide two benefits with cell replacement treatment in PD: (1) differentiation into broad spectrum of cells for replenishing lost DA neurons and (2) trophic effect that is mediated by various types of trophic factors^18^. MSCs, upon transplantation into brain, promote neuronal growth, decrease apoptosis, regulate inflammation and modify the niche to increase neural regeneration. Their mode of action includes release of trophic factors which further induce survival and regeneration of host neurons^19^. There are several experimental cell replacement studies reporting the efficacy and neuro-regenerative and neuro-restorative potential of MSCs in PD rat models and in clinical trials^20,21^. MSCs have also been explored as delivery vehicles for the production of additional factors aiding neuronal growth at the site of injury. MSCs have also been used in several US FDA-approved clinical trials for myocardial infarction, stroke, meniscus injury, limb ischemia, graft-versus-host disease and autoimmune disorders^22^. However, there are certain acute issues which need to be addressed, like (a) patient selection, (b) site of administration of cells, (c) parameters for cell preparation and delivery in terms of optimizing graft survival, dosage and format of cells, and (d) optimizing graft function and preventing GvHD^23^. Hence, our aim was to investigate the effect of 22-HC on hMSCs, which in turn have the most optimum translational implication. Considering the specific action of 22-HC in DAergic neurogenesis and very few explanatory studies using hESCs or in vivo mice models, we ventured to explore its contribution towards generation of DA neurons from hMSCs and the regenerative effect of hMSCs (if any) in PD Wistar rat model. The study also reports the use of in vitro coaxed MSCs by 22-HC in PD animal model. This detailed comparative analysis on one platform has given the futuristic direction of research in the field of DAergic neurogenesis and regenerative medicine.

## Results

### hMSCs require higher concentration of 22-HC for differentiation into neuronal cells

A higher dose of 22-HC is required to differentiate hMSCs into DAergic neuronal cells. As per the previously reported dose of 22-HC (0.5 μM–1.0 μM) with hESC lines^5^, maximum neurogenic effect was observed at a concentration of 0.5 µM of 22-HC. We titrated the dose of 22-HC on BM-MSC from a range of 0.5–3.0 µM and evaluated the results by flow cytometric enumeration of MAP2 and TH-positive cells to use 2 µM of 22-HC for further experiments.

### 22-HC causes neuron-like morphological changes in the hMSCs

Morphological changes were observed after treating hMSCs with 22-HC and FGF2. Spindle-shaped morphology of MSCs changed to more neuronal one with distinct cell body, perikaryl nucleus and cytoplasmic extensions like neurites and axon. The terminals of the induced cells also had multiple dendritic structures (Fig. 1.1), as confirmed by scanning electron microscopic (SEM) studies. Several fields showed cell to cell interaction, with extended neurites like extensions. Axon-hillock-like structures were also observed in the differentiated neuronal cells (Fig. 1.2 A i and ii).

**Fig. 1:**
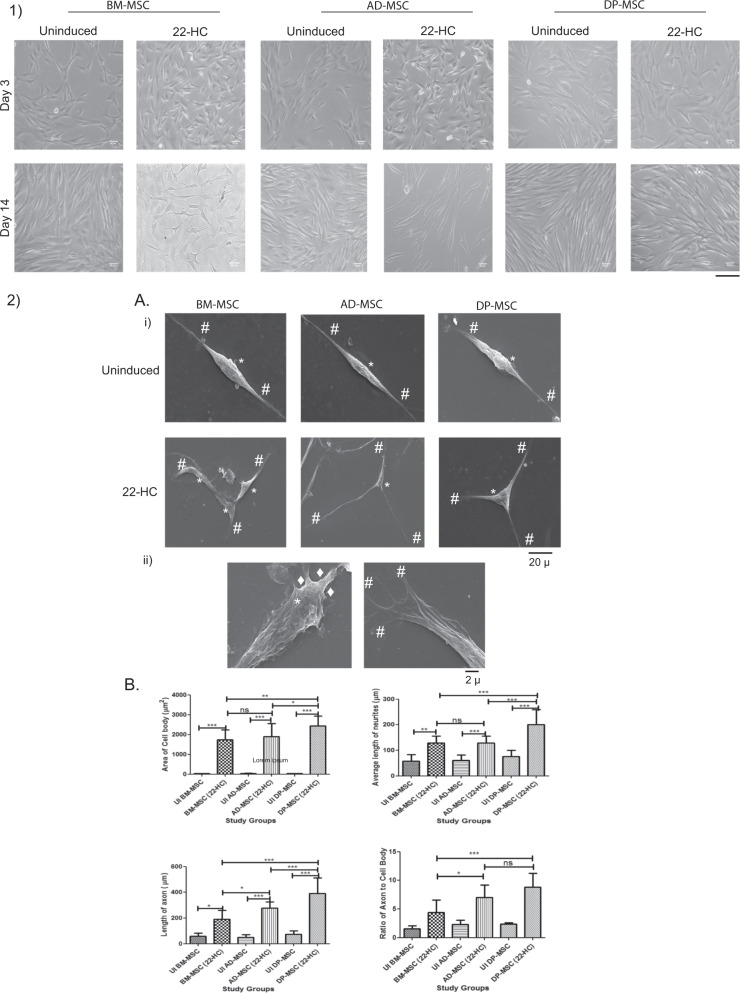
1: Morphological changes occurring in hMSCs during various time points (day 3, day 5, day 10 and day 12) of neuronal induction. (i) BM-MSC, (ii) AD-MSC and (iii) DP-MSC. The morphology of hMSCs has changed from spindle shaped to perikaryl. Appearance of neuronal morphology starts appearing from 6 to 7th day of induction (scale bar 100 µm). 2: Morphological and Morphometric Characterization of differentiated hMSCs: **A** Scanning electron microscopic observations depicting minute morphological changes occurring in hMSCs after neuronal induction (i) morphology of hMSCs has changed from spindle shaped to perikaryl. Terminals of the cells show appearance of minute neurites’-like structures, which enhance cell-cell interactions. Here, # denotes neuritis, *denotes cell body and •denotes axon hillock (Scale bar 20 μm). (ii) magnified images of differentiated cells, showing the appearance of axon-hillock, neuritogenesis and appearance of terminal neurites, facilitating cellular responses and interactions (Scale bar 2 μm). **B** Morphometric analysis of the neuronal cells generated in vitro by using 22-HC (i) area of the cell body under various study groups. The graph depicts significant difference in this parameter between AD-MSC and DP-MSC; (ii) average length of neurites of differentiated cells after induction. The graph shows that the difference in the length of neurites is significantly higher in differentiated DP-MSCs, as compared to that in BM-MSCs and AD-MSCs; (iii) length of axons of cells after neuronal induction. The graph shows that the difference in the axonal length follows similar trend as average length of neurites; (iv) ratio of axon to cell body of cells under various study groups. The graph depicts that this morphological parameter of neuronal cells shows significant difference among all the three types of hMSCs under the study. Data points represent the means ± SD (*n* = 3), *p < 0.001 vs. control untreated cells. For all the parameters under the study, five different samples of each type of hMSC were taken (*n* = 40 for each study group). Data was analysed by three independent observers.

### 22-HC induces neuronal cells-like features in hMSCs with increase in the average area of cell body, length of neurites and axon

Average of the cell body increased from 38.51 ± 0.8 to 1737 ± 116.3 µm^2^ in BM-MSCs, from 43.03 ± 3.0 to 1897 ± 130.1 µm^2^ in AD-MSCs and from 37.85 ± 1.5 to 2430 ± 119.7 µm^2^ in DP-MSCs. Average length of the neurites increased from 57.59 ± 11.4 to 129.1 ± 6.2 µm in BM-MSCs, from 61.49 ± 6.7 to 128.7 ± 5.5 µm in AD-MSCs and from 75.26 ± 10.9 to 199.8 ± 13.5 µm in DP-MSCs. An upsurge from 57.59 ± 11.4 to 191.8 ± 16 µm in BM-MSCs, from 49.09 ± 6.9 to 278.5 ± 10.4 µm in AD-MSCs and from 75.26 ± 10.9 to 390.5 ± 25 µm in DP-MSCs was observed with axon length. No significant difference was found in the area of cell body and average length of neurites of induced BM-MSCs and AD-MSCs. On the contrary, length of axon and ratio of axon to cell body was found to be significantly higher in induced AD-MSCs as compared to those in induced BM-MSCs. DP-MSCs consistently had higher escalation in all the studied parameters (Fig. 1.2 B i–iv).

### 22-HC leads to upregulation of DA neuronal cell traits at transcriptional level in hMSCs

**Fig. 2:**
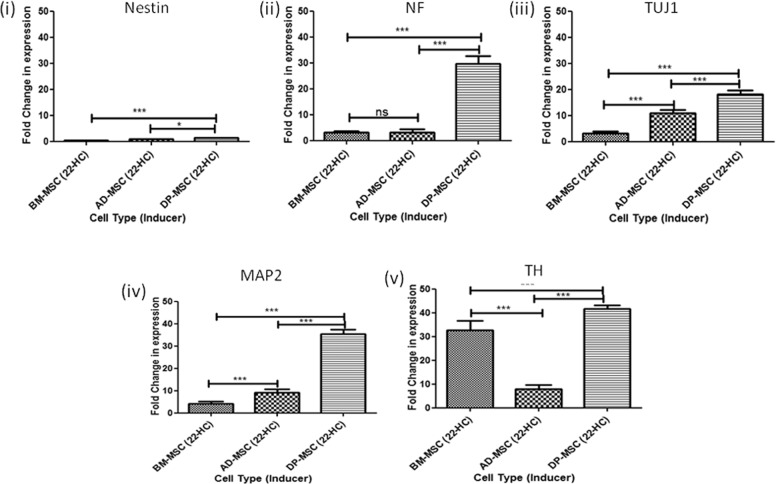
qRT-PCR transcriptional analysis of differentiated hMSCs. (i) Relative fold change in mRNA expression of nestin in induced hMSCs, as compared to respective uninduced counterparts; (ii) relative fold change in mRNA expression NF in induced hMSCs, as compared to respective uninduced counterparts; (iii) relative fold change in mRNA expression of TUJ1 in induced hMSCs, as compared to respective uninduced counterparts; (iv) relative fold change in mRNA expression of MAP2 in induced hMSCs, as compared to respective uninduced counterparts; (v) relative fold change in mRNA expression of TH in induced hMSCs, as compared to respective uninduced counterparts. Data points represent the means ± SD (*n* = 3), *p < 0.001 vs. control untreated cells.

Post induction, a relative upregulation was observed in the expression of NF (3.16 ± 0.2, 3.3 ± 0.4 and 29.6 ± 1.3 folds in BM-MSCs, AD-MSCs and DP-MSCs, respectively), TUJ1 (3.1 ± 0.2, 10.9 ± 0.5 and 18.06 ± 0.6 folds in BM-MSCs, AD-MSCs and DP-MSCs, respectively), MAP2 (4.07 ± 0.45, 9.07 ± 0.68 and 35.3 ± 0.90 folds in BM-MSCs, AD-MSCs and DP-MSCs, respectively) and TH (32.6 ± 1.7, 7.8 ± 0.8 and 41.6 ± 0.7 folds in BM-MSCs, AD-MSCs and DP-MSCs, respectively). However, no significantincrease was observed in mRNA expression of nestin, except that in DP-MSCs with 1.3 ± 0.2 folds (Fig. 2). Mature neuronal markers, TUJ1 and MAP2 followed a similar trend, with significantly higher upregulation in AD-MSCs as compared to that in BM-MSCs.

### 22-HC causes increase in the DA neuronal cell-specific proteins, corresponding to their transcriptional expression

**Fig. 3:**
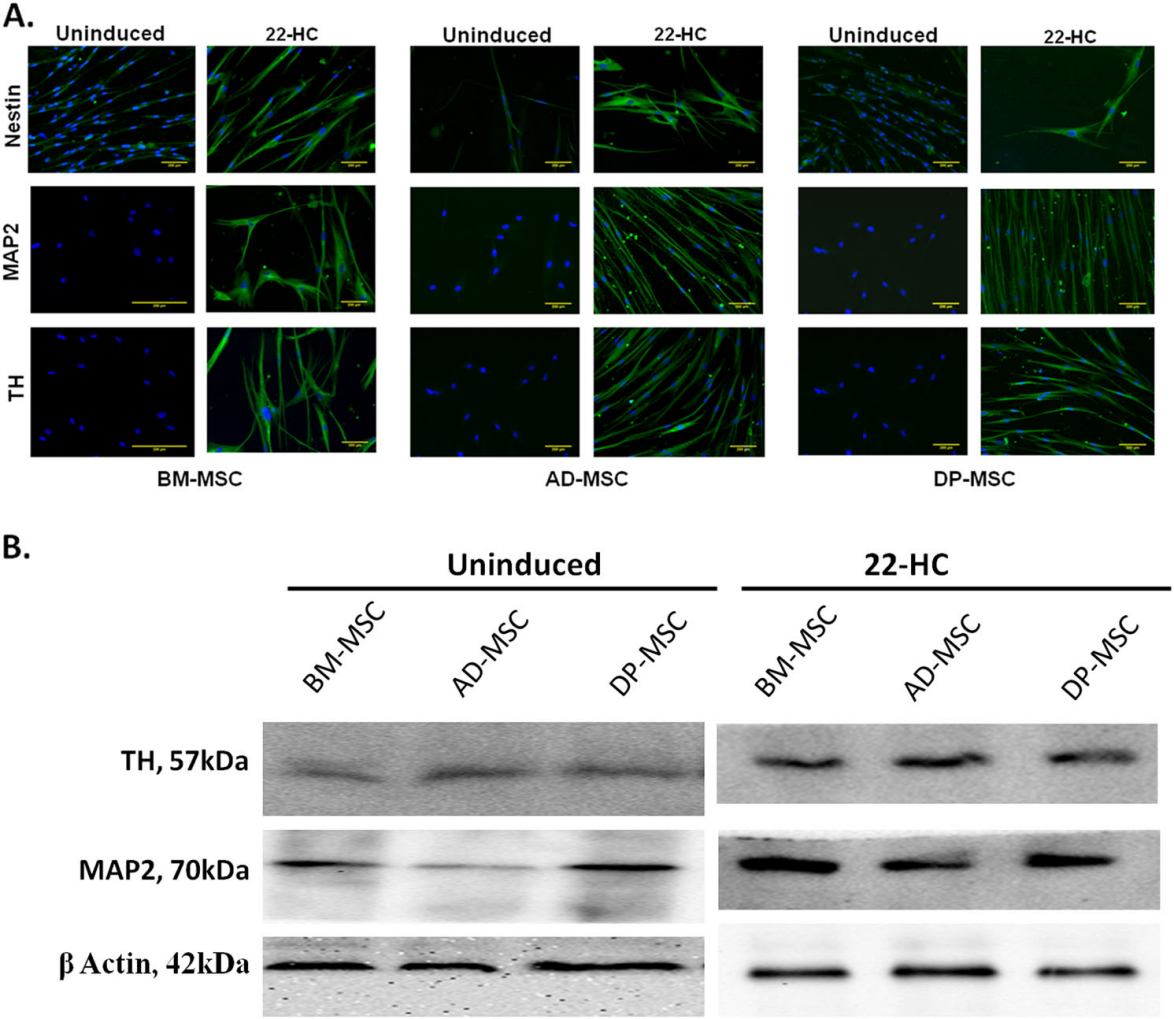
Expression of neuronal cell-associated proteins in differentiated hMSCs. **A** Immunoflorescence assay showing expression of nestin, MAP2 and TH protein expression in hMSCs pre-differentiation and post-differentiation into DAergic neuronal cells. **B** Immunoblotting assay for expression of neuronal and DA neuronal cells associated proteins (MAP2 and TH) in uninduced and differentiated hMSCs.

There was only basal level of expression of MAP2 and TH in uninduced hMSCs, while a higher expression was observed post-induction in all the hMSC types (Fig. 3). Increased fluorescence intensity in the images supports higher expression of MAP2 and TH in differentiated cells. However, among the various hMSCs types, the highest fluorescence intensity of these protein markers was observed in case of DP-MSC. Similar trend was also observed when immunoblotting assay was performed in both uninduced and induced groups (Fig. 3).

### Induction of hMSCs using 22-HC increases the percentage of cells positive for DAergic neuronal proteins

**Fig. 4:**
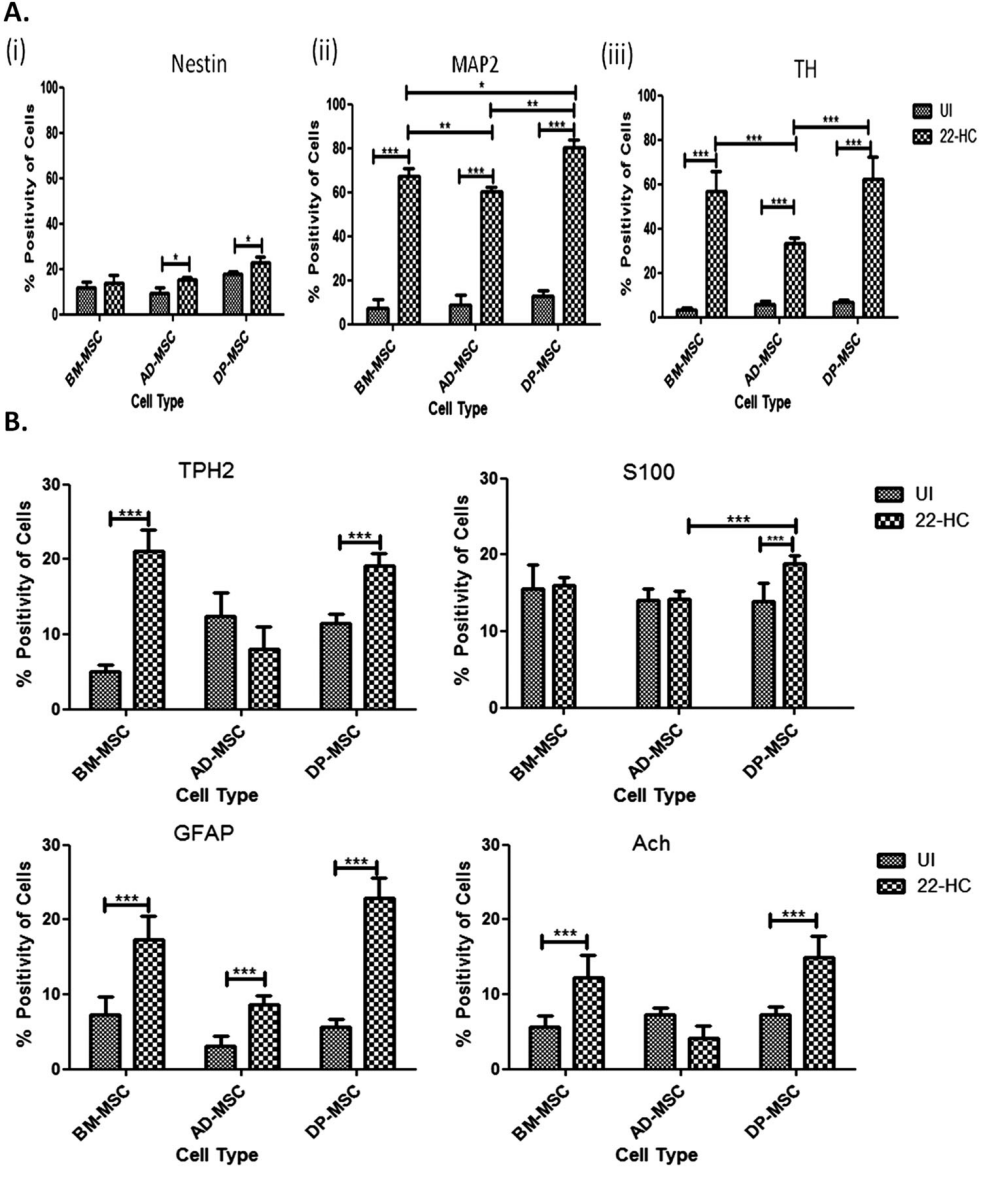
Flow cytometric analysis for cell enumeration. **A** (i) Graph depicting the number of nestin-positive cells pre-neuronal and post-neuronal induction. DP-MSC have maximum number of nestin positive (p*), cells at the beginning of experiments, followed by those in AD-MSC and BM-MSC; (ii) graph depicting the number of MAP2-positive cells pre- and post-neuronal induction. (p*); (iii) graph depicting the number of TH-positive cells pre- and post-neuronal induction. Data points represent the means ± SD (*n* = 3), *p < 0.001 vs. control untreated cells. **B** Flow cytometric analysis for cell enumeration of non-DAergic neuronal cells in the cell-milieu, pre-neuronal and post-neuronal differentiation of hMSCs.

Except in BM-MSCs, nestin-positive cells were increased upon differentiation (from 9.4 ± 0.9% to 15.1 ± 0.5% in AD-MSCs and from 17.8 ± 0.5% to 23 ± 1.1% in DP-MSCs). MAP2-positive cells were significantly increased post induction in all hMSC types. Maximum increase was observed in DP-MSCs (80.20 ± 1.5%), followed by that in BM-MSCs (67.3 ± 1.5%) and AD-MSCs (60.1 ± 0.9%) (Fig. 4A (i) and (ii)). No marked difference was observed in the percentage of cells positive for TH between BM-MSCs and DP-MSCs post-neuronal induction (56.9 ± 4.0% and 62.3 ± 4.5%, respectively) (Fig. 4A (iii)).

### Cell milieu of the induced hMSCs: presence of non-DA neuronal cells

Cell milieu of the induced hMSCs consisted of cells positive for non-DAergic proteins like TPH2, S100, GFAP and Ach. The percentage of TPH2 increased significantly in BM-MSC and DP-MSCs only; while it decreased in AD-MSCs (however non-significantly). Similar trend was observed with Ach-positive cells. However, cells positive for S100 increased only in DP-MSCs post-induction (Fig. 4B).

### Upregulation of the gene expression of transcription factors responsible for maturation and survival of DAergic neurons

**Fig. 5:**
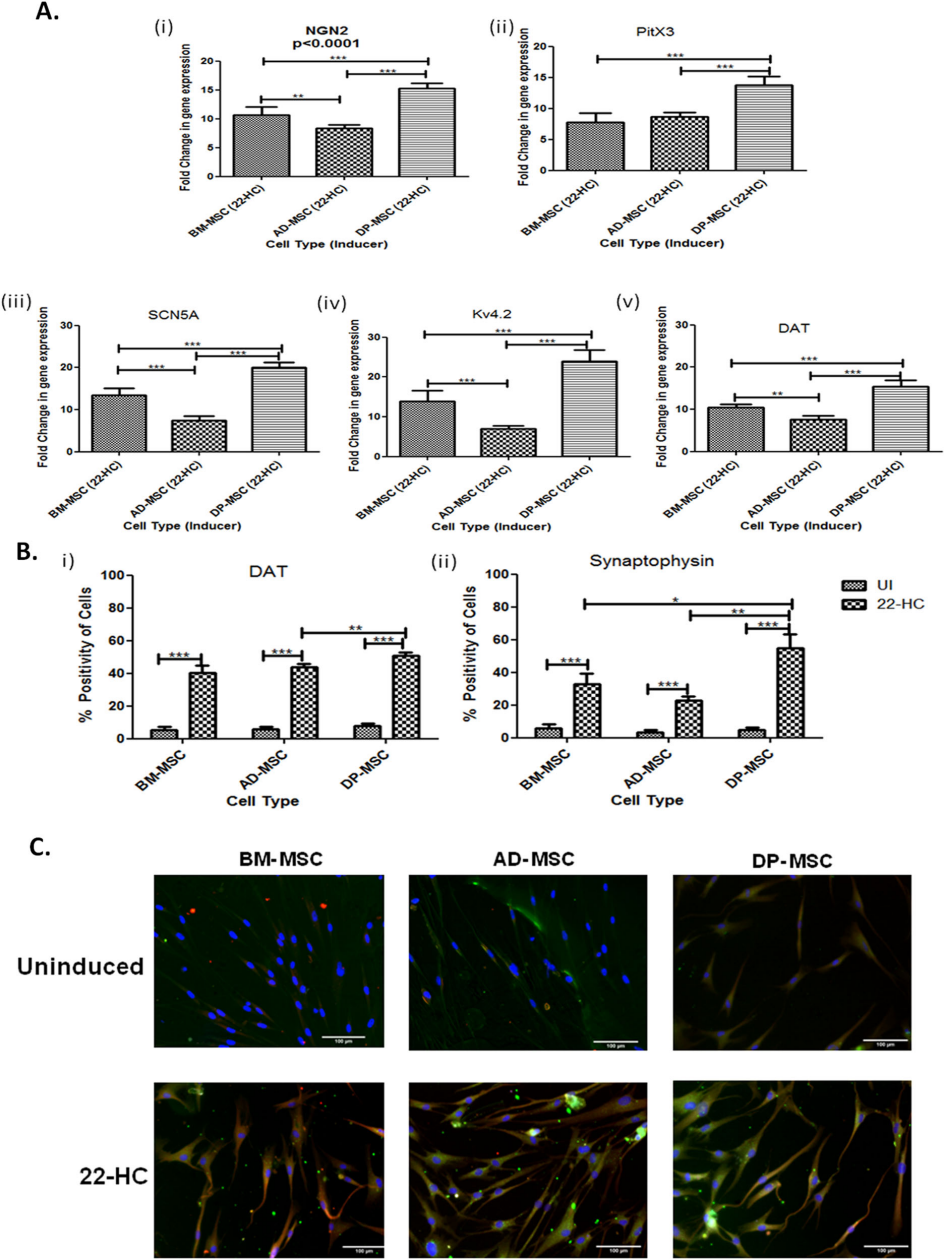
Assessment of genes and proteins associated with neuronal functionality. **A** qRT-PCR mRNA transcriptional analysis of differentiated hMSCs for genes associated with functional behaviour of DAergic neuronal cells. Data points represent the means ± SD (*n* = 3), *p < 0.001 vs. control untreated cells. **B** Flow cytometric enumeration of number of cells positive for (i) dopamine transporter protein, a protein responsible for release of dopamine neurotransmitter through vesicles and (ii) synaptophysin protein which is responsible for the formation of synapse between two neuronal cells. Data points represent the means ± SD (*n* = 3), *p < 0.001 vs. control untreated cells. **C** Immunoflorescence assay to show the expression of TH and DAT proteins in hMSCs pre-differentiation and post-differentiation into DAergic neuronal cells.

22-HC also increased the expression of transcription factors, which are responsible for maturation and survival of DAergic neurons^12^. While BM-MSCs and AD-MSCs showed no significant difference in the expression of PitX3, yet remarkable increase in the expression of NGN2 in BM-MSCs (10.6 ± 0.6 folds) as compared to those in AD-MSCs (8.3 ± 0.3 folds) was seen. Whereas, DP-MSCs maintained the trend with highest increase in gene expression (13.75 ± 0.6 folds of PitX3 and 15.3 ± 0.4 folds of NGN2 in BM-MSCs, AD-MSCs and DP-MSCs, respectively). These results show that the differentiated hMSCs expressed prototypical midbrain DAergic markers at transcriptional level (Fig. 5A (i) and (ii)).

### 22-HC improves functional DAergic specifications at both gene and protein levels

A constant trend was observed where maximum increase in the expression of three genes (15.34 ± 0.3 folds DAT, 24.02 ± 0.56 folds Kv4.2 and 20.12 ± 0.63 folds SCN5A) was highest in DP-MSCs, followed by that in BM-MSCs (10.5 ± 0.3 folds DAT, 13.9 ± 1.1 folds Kv4.2 and 13.4 ± 0.7 folds SCN5A) and AD-MSCs (7.8 ± 0.4 folds DAT, 6.9 ± 0.4 folds Kv4.2 and 7.5 ± 0.4 folds SCN5A). Extent of escalation of these functionality related genes was observed to be significantly lower in differentiated AD-MSCs, as compared to that in other hMSC types (Fig. 5B (iii)–(v)).

The number of cells in differentiated hMSCs positive for DAT and synaptophysin was significantly higher than that in naive MSCs. While no denoting difference was observed between the outcome of BM-MSCs (40.2 ± 2.0%) and AD-MSCs (43.8 ± 0.9%) for DAT-positive cells, DP-MSCs showed positively higher percentage of DAT-positive cells (50.8 ± 0.9%). Likewise, synaptophysin-positive cells were found to be highest in number in differentiated DP-MSCs (54.9 ± 3.7%), followed by that in BM-MSCs (33.0 ± 2.7%) and AD-MSCs (22.8 ± 1.1%). The difference was significant among all the induced cell types (Fig. 5B (i) and (ii)).

**Fig. 6:**
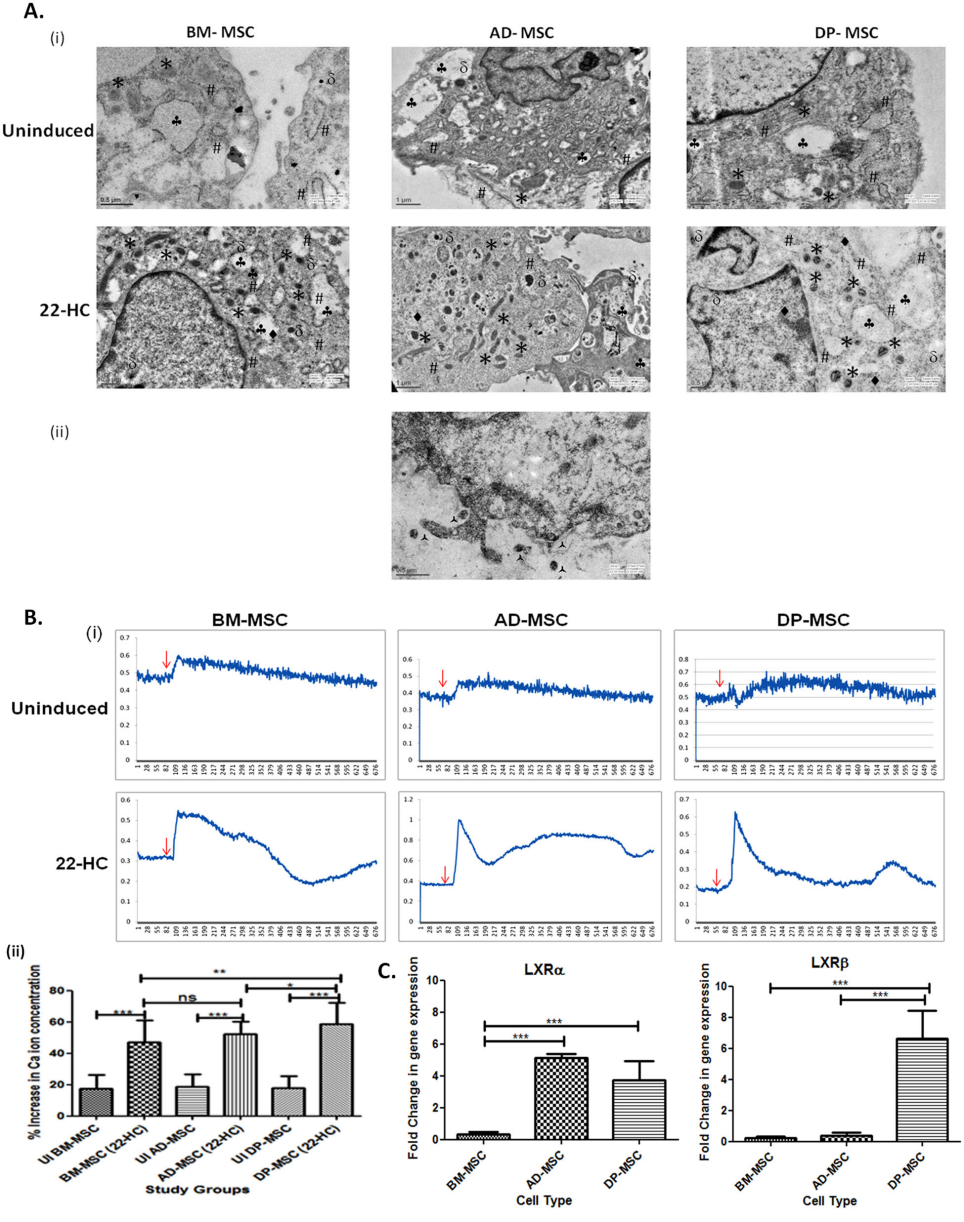
Functional Characterization of hMSCs. **A** Transmission electron microscopic observations depicting ultra-structural changes occurring in hMSCs after neuronal induction. (i) Ultra-structural composition of hMSCs has changed on various parameters. There was observed increased mitogenesis, increase in dense core vesicles, rough endoplasmic reticulum, cytoskeletal condensation and endocytotic vesicles. The genesis of these cellular organelles may be associated with the increased functionality of the terminally differentiated hMSCs. (ii) Part of the cell membrane showing the release of vesicles (probably synaptic vesicles) by exocytosis, contributing to the chemical functionality of the DAergic neuronal cells. In this figure, # represent rough endoplasmic reticulum, * represent mitochondria, represent endocytotic vacuoles, • represent cytoskeletal condensation and represent dense core vesicles. å represent the exocytotic vesicles released from differentiated DP-MSCs. **B** Calcium ion imaging analysis by Fura red-AM ratiometric dye: (i) line graphs showing the changes in the Ca^2+^ transients upon depolarization with KCl in cells of all study groups. The uninduced hMSC did not show any change in the intracellular Ca^2+^ concentration after depolarization. The red arrows indicate the time point of addition of KCl for depolarization in the cell culture; (ii) graph showing the change in the percentage increase in the calcium ion concentration in hMSCs obtained from various tissue sources after DAergic neuronal induction by 22-HC. Data points represent the means±SD (*n*=5), *p<0.001 vs. control untreated cells. **C** qRT-PCR transcriptional analysis of liver X receptors α and β, which are the main nuclear receptors, reported to date, responsible for DAergic neurogenesis by oxysterols (22-HC). Our results suggest a prominent role of LXRβ in DAergic neurogenesis with 22-HC as inducer DP-MSC only. LXRα does not seem to play much distinct role in this process. However, least upregulation of both of these genes was observed in BM-MSCs.

Furthermore, immunoflorescence staining revealed the presence of both TH and DAT proteins in all the hMSC types post induction with very little or no expression in naive hMSCs (Fig. 6C).

### Ultra-structural changes, contributing towards functional DA neuronal cell specification were observed

Neuronal differentiation of hMSCs is associated with the ultra-structural modifications in the mitochondria^13^, dense core vesicles or granules (DCVs)^24^, rough endoplasmic reticulum (RER)^25^, cytoplasmic filamentous condensation^25,26^ and endocytic vesicles^26^.

Mitochondrial biogenesis was increased in the differentiated DAergic neuronal cells, as is evident by the increase in the number of mitochondria, with globular cup-like structure and evident cristae. (Fig. 6A).

The number of DCVs and RER was increased, attributing towards the increased functionality of the neuronal cells, which is an indicative of the increased functionality of the cells (Fig. 6A). Cytoskeletal condensation is an indicative of neuritogenesis and axonogenesis and was observed in all types of hMSCs post differentiation. Microtubules were observed to be arranged in a synchronized pattern after differentiation of hMSCs with 22-HC. Small endocytotic vesicles were also observed in the ultra-structural study of hMSCs post-induction (Fig. 6A).

### hMSCs show increase in the calcium ion efflux upon treatment with 22-HC

In DP-MSCs the change in calcium ion transients was observed to be maximum (58.5 ± 2.6%) as compared to the control (17.9 ± 1.7%). This was followed by that in AD-MSCs (52.1 ± 1.8% in differentiated and 18.7 ± 1.7% in control) and least calcium ion transients were observed in BM-MSCs (46.98 ± 2.566% in differentiated and 17.5 ± 1.9% in control). The change in the calcium ion concentration in various study groups have been detailed in Fig. 6B (i) and (ii).

### Liver X receptors (α and β) acknowledge 22-HC differently with different types of hMSCs

AD-MSCs showed highest upsurge of LXRα (5.1 ± 0.1 folds), followed by that in DP-MSCs (3.7 ± 0.6 folds), with almost negligible changes in case of BM-MSCs. On the contrary, LXRβ was maximally expressed in DP-MSCs (6.6 ± 0.9 folds), followed by that in AD-MSCs and BM-MSCs, with negligible upregulation (Fig. 6C). This difference in the expression of both the receptors in different hMSC types may be attributed to their origin, chief function(s) in the living system and pathway(s) that might have been activated, resulting in DAergic neurogenesis.

### Testing the therapeutic effect of hMSCs in Parkinsonian rats: animal work results

**Fig. 7:**
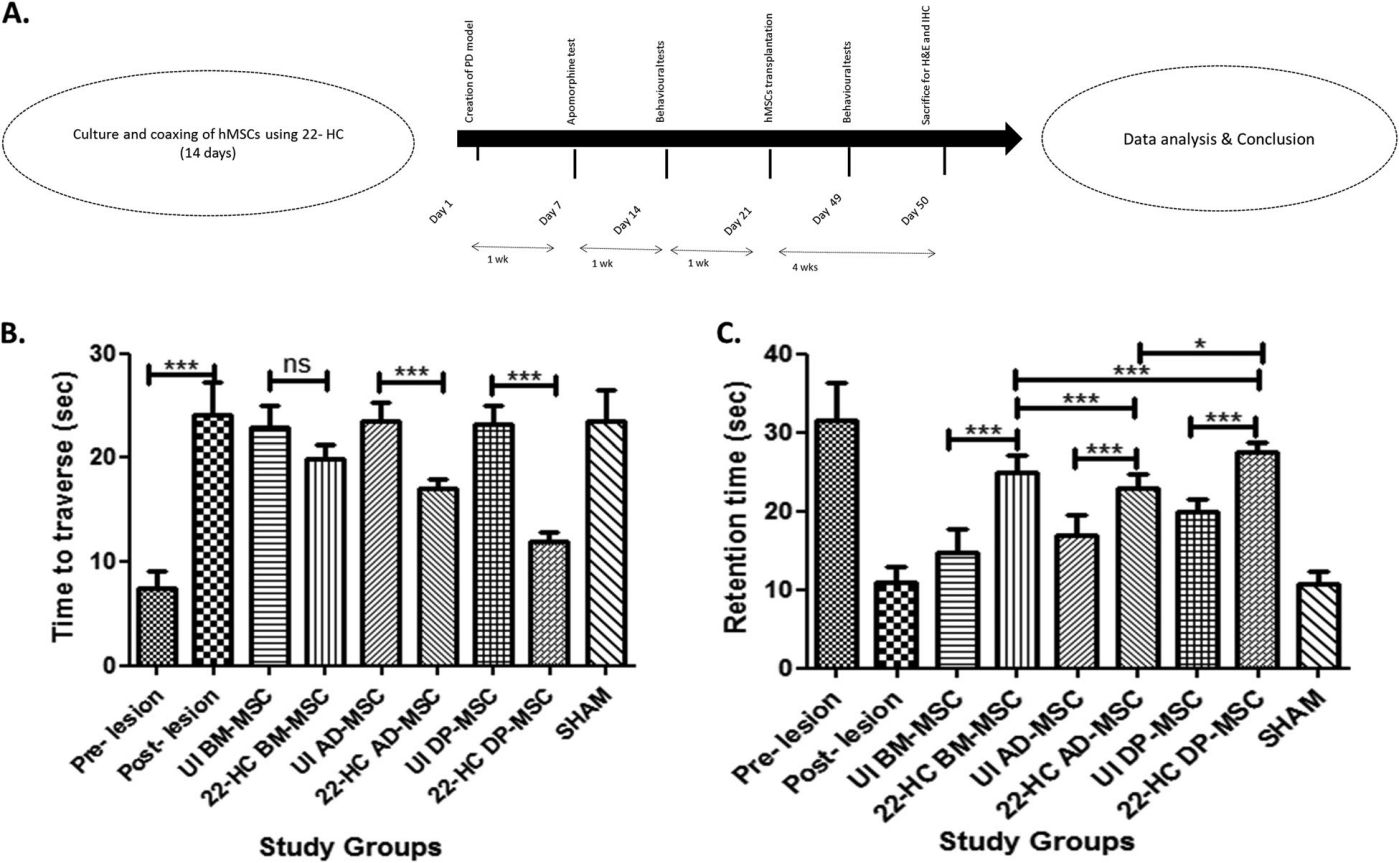
Validation of in vitro results in Wistar rat PD models. **A** Schematic representation to show the sequence of in vivo stem cell transplantation experiments. **B** Balance beam test: restoration of motor deficit function in rats who received either naïve or differentiated hMSCs. While the motor function in all the rats was reduced significantly after creation of lesion by 6-OHDA, it was reduced further significantly upon hMSCs transplantation, with maximum effect in case of differentiated DP-MSCs. This test was performed 4 weeks after transplantation of hMSCs. Data points represent the means±SD (*n*=5), *p<0.001 vs. control untreated cells. **C** Rotarod test: The retention time of the rats was increased significantly after transplanting them with hMSCs in naïve or differentiated form. However, the restoration of motor neuron function was the most in case where differentiated DP-MSCs were used.

The experiments were performed as per the schematic illustration given in Fig. 7. After surgery, the food and water intake of the rats were monitored and observed, along with any signs of infections. There was a reduction of food and water intake by the rats, resulting in reduction in a loss of the body weights (data not shown). However, the reduction was not observed after second week of the surgery.

**Fig. 8:**
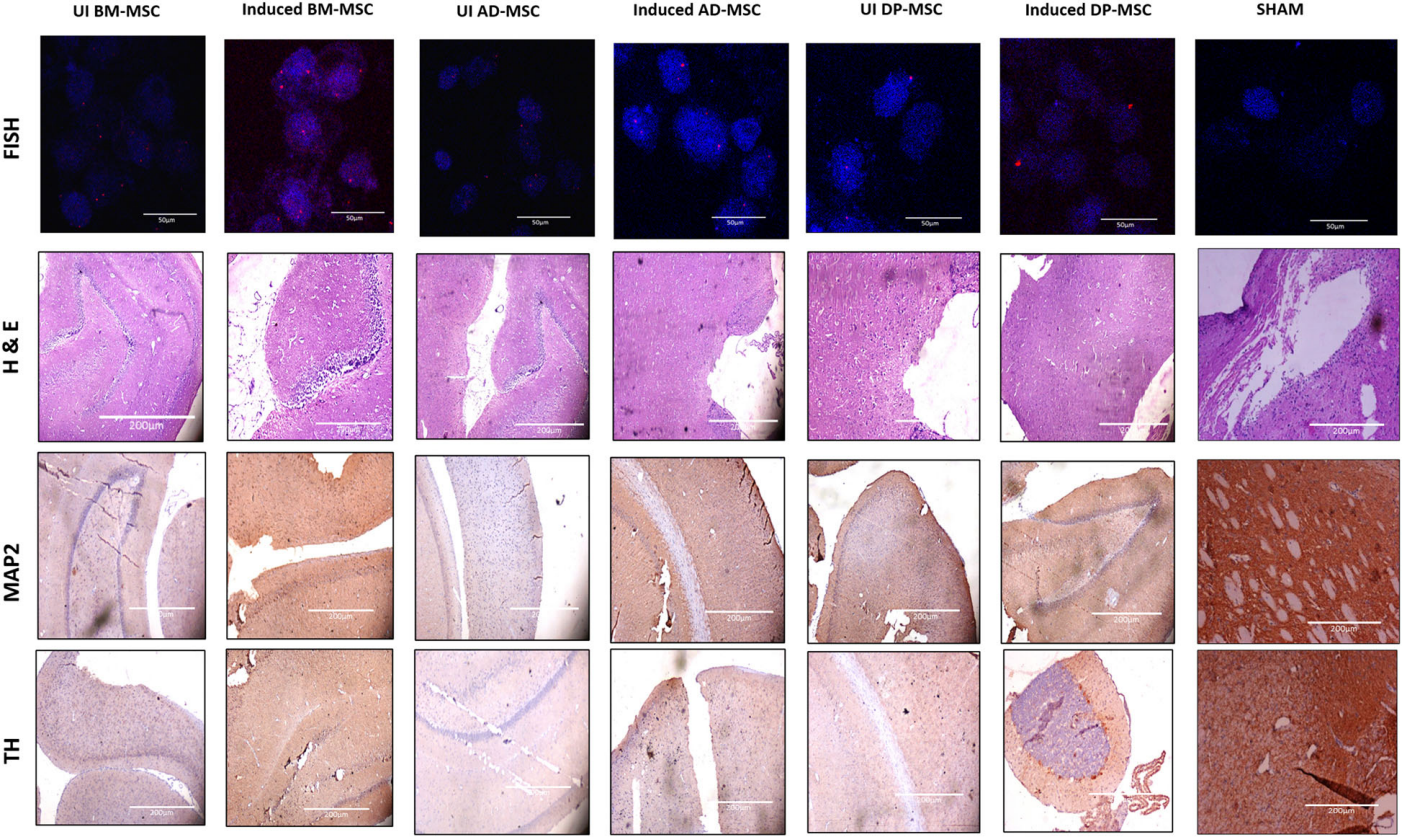
Fluorescent in situ hybridization study. To investigate the homing of transplanted hMSCs in the SNpc region of Wistar rat brain (scale bar 50 µm); Hematoxylin and eosin staining to check the presence of any inflammatory cells in the SNpc region of Wistar rat brain (scale bar 50 µm); immuno-histochemical analysis to study the differential expression of MAP2 and TH proteins in the SNpc region of Wistar rat brain (scale bar 200 µm). All these analyses were performed subsequently in all the study groups.

After one week of surgery, the rats with more than 60 rotations per 30 min were selected for further experiments, based on the apomorphine test performed. To further confirm the induction of neurodegeneration on the rats, their behavioural changes were analysed for APO-induced rotation, motor coordination and balance skills after 2 weeks. Prior to performing behavioural studies, homing of MSCs were ensured by FISH analyses in which the part of rat brain where MSCs were transplanted was taken and processed for X-linked probing for human chromosomes. Categorically, hMSCs from female donors were used to be transplanted in the male Wistar rats to avoid any false positive results during homing studies. We observed that there was a presence of X probe positive cells as well as cells which showed absolute absence of any probes, proving that the brain tissue under analysis contained cells of both human and rat origin even after 2 weeks of transplantation (Fig. 8).

Behavioural studies were performed 2 weeks post transplantation of hMSCs. Rotarod results revealed that the retention time of rats post-surgery reduced significantly from an average of 31–10 s. Coaxed and naïve MSCs (1 × 10^5^ per 4 µL PBS) were transplanted after 2 weeks of lesion surgery. The retention time increased significantly in all the groups where coaxed hMSCs were transplanted, as compared to their naïve counterpart hMSCs, highest retention time being with coaxed DP-MSCs (~28 s), followed by that in BM-MSCs (~25 s) and AD-MSCs (~21 s), with significant difference. Similar trend was observed with beam test. While the time taken to traverse the beam increased post-surgery, it was reduced significantly upon transplanting hMSCs. As compared to the naïve hMSCs, coaxed hMSCs yielded significantly better recovery of motor functions in rats. In compliance with the results of rotarod test, recovery of motor functions was better with DP-MSCs with traverse time of ~12 s, followed by that with AD-MSCs (~17 s) and BM-MSCs (~20 s) (Fig. 7).

Upon careful examination of the sections, it was observed that the presence of inflammatory cells was almost nil in the transplanted region. Also no prominent signs of necrotic neurons were observed. Immunohistochemical analysis revealed that while the expression of MAP2 and TH decreased in the lesion region of rat brain, it increased in the groups where hMSCs were transplanted. However, the expression of both the proteins was higher in groups transplanted with coaxed hMSCs. The results with DP-MSCs were corresponding with the previous experiments and observations (Fig. 8).

## Discussion

There had been no reports suggesting the role of oxysterols in differentiation of hMSCs. However, one study demonstrated the role of oxysterols and their nuclear receptors, LXRα and LXRβ in human midbrain DAergic neurogenesis^5^. This study was performed on mouse embryo and hESCs. It was observed that after adding 22-HC in the induction cocktail, the percentage of TH-positive cells was increased from 22% to 60% with the subsequent decrease in GFAP-positive cells. Thus, the study reports the usage of 22-HC for differentiating hESCs in vitro and prospects their use in regenerative medicine and drug testing in future^5^.

However, using hESCs in clinical set up is not recommended by several researchers and clinicians due to their immunogenicity and tendency to form teratoma. Hence, MSCs are preferred for both clinical and drug testing purposes. In our current study, we have, for the very first time, investigated the effect of 22-HC on DAergic neuronal differentiation of human MSCs, derived from human BM, AD and DP. The investigation included detailed morphological, morphometric, transcriptional, translational, ultra structural and functional characterization of DAergic neuronal cells derived from hMSCs.

We hereby report the use of 22-HC as a novel inducer for efficient in vitro generation of DAergic neuronal cells from hMSCs, and their detailed multi-factorial characterization. Our protocol reports as high as 80% MAP2-positive cells after induction of DP-MSCs. This is the highest percentage of in vitro-generated mature neurons reported till now^14,27–31^. Our protocol also yields the maximum number of TH-positive DAergic neuronal cells (72% in case of DP-MSCs)^5,7,10–12,32^. To the best of our knowledge, these statistics are the highest among all the reported studies to date. DP-MSCs showed maximal efficiency of in vitro generation of DAergic neuronal cells, followed by BM-MSCs and least being in AD-MSCs. The protocol presented is not only the most efficient, but also cost-effective, as it includes the use of only two inducers for generation of DAergic neuronal cells in vitro from hMSCs for translational purpose.

We, for the very first time, report the ultra-structural changes occurring in hMSCs upon in vitro differentiation into DAergic neuronal cells. While we have commented on the fine structural changes that appear upon differentiation of hMSCs into neuronal cells, we have also studied in detail the metamorphosis occurring in the cellular components after differentiation.

The study also hints that LXRβ plays a prominent role in DAergic differentiation of hMSCs as compared with LXRα. However, further detailed investigations are required to prove this hypothesis. Also, the noteworthy observations in the difference of transcriptional expression of LXRα in case of AD-MSCs may be reasoned with their constitutive function of fat storage and synthesis of oestrogens and androgens^33^ in the living system. We hypothesize that LXRα is more upregulated in AD-MSCs, to activate their default pathway(s). As no direct or indirect proof is available to state the exact role of these two receptors in DAergic neurogenesis and differential effect on various types of embryonic or adult stem cells, our hypothesis, needs further experimental validation.

MSCs obtained from various sources have been explored for their engrafting ability, homing capacity, migration, and regeneration potential in various neurodegenerative diseases^34,35^. There are two theories related to the transplantation of MSCs for neuro- regeneration. First one states that naive MSCs differentiate into neurons with the help of cues present at the site of transplantation in the brain. Second theory suggests coaxing of MSCs into neuronal cells before transplantation. Hence, in the current study we studied the differential neuroregenerative potential of naive and differentiated hMSCs upon stereotaxic transplantation in PD Wistar rat models.

Through the in vivo experiments, we observed that the donor hMSCs engrafted and homed well in the transplanted area of the brain. We opted for transplanting hMSCs in the SNpc region only as there were previous reports stating inefficiency of intravenous injection of stem cells in animals due to non-retention of stem cells in the desired brain area^36^. Results obtained from motor functional studies were further validated by apomorphine-induced rotation, causing bilateral imbalance of the nigrostriatal systems^37,38^. In control rats, no such movements were observed because of bilateral balance of the nigrostriatal systems. Substantial decrease in the contralateral rotation of the treated compared with sham control rats confirmed that bilateral balance of the nigro-striatal systems was partly established by the presence of functional DA receptors of the donor-derived cells. This was confirmed by FISH analyses of the tissue from the region where cells were transplanted. While human X-probes could be observed in most of the cells, there were also observed cells with no signals from human X-probe. These were the cells of rat origin, which came along with the extracted tissue from the rat brain. The results of the behavioural assessment indicated an improvement in the motor functions of the transplanted PD Wistar rat model, performed 4 weeks after hMSC transplantation. However, a long-term study is justified to validate our present findings. Immuno-histochemical analysis results also confirmed the generation of DA cells in the area of transplanted MSCs. These results indicate that neuronal coaxed hMSCs have better neuro-regenerative potential as compared to that of naive, as no noteworthy improvement was observed with naive hMSCs. In earlier studies^27,39^, MSCs were transplanted within 7 days of injury, and the unhealthy host neurons were protected by donor-derived neurotrophic factors. The recovery in motor function was not equivalent to that reported in similar studies^39,40^. These differences could be attributed to the nature and number of cells transplanted and responses within the experimental animals. Similar kind of studies have been conducted in the past^38,40^. Most of these studies targeted the use of naïve hMSCs for transplantation^26,36,41^. However, there were a few reports stating the transplantation of coaxed stem cells^28,38,42,43^. Our current study aims the use of human tissue-derived MSCs unlike several other studies where they were derived from rats or mouse. It gives a more reliable picture of the use of hMSCs in treating neurodegenerative diseases. Another salient feature of our study is the use and comparison of hMSCs from different tissue sources. This is one of its own kinds of study where this type of comparison has been reported from the core. It gives a wholesome picture of which tissue sources may be targeted for neurodegenerative diseases.

In conclusion, our study provides comprehensive and robust evidence to state the role 22-HC in generating functional DAergic neuronal cells from hMSCs of varied origins. These in vitro-generated cells showed DAergic neuronal specifications with tremendous potential in clinical and pharmacological applications. As reported earlier^5^, functions of LXRs are conserved in human cells. Our study gives the first evidence that oxysterols (22-HC) causes DAergic differentiation and leads to several changes in hMSCs. Our research has also paved the path to investigate the disparate effect of 22-HC on the three types of hMSCs taken under the study. AD-MSCs have shown off the beat expression of LXRα as compared to that of LXRβ. These pathways need to be investigated further to have an insight of the mode and mechanism of action of 22-HC for midbrain DAergic neurogenesis. This study further testifies that differentiated hMSCs have better neuroregenerative potential than that of naive hMSCs as depicted by the behavioural and IHC assessment of the rats. The current study gives an inclination towards future prospects to be explored in the area of stem cells and neuro-regeneration.

## Materials and methods

### Ethics statement

The study was conducted after receiving ethical clearances from Institutional Committee for Stem Cell Research (IC-SCR) (Ref. No. IC-SCR/37/15(R)) and Institutional Animal Ethics Committee (IAEC) (Ref. No. 955/IAEC/16), AIIMS, New Delhi. All the methods described in this study were performed in accordance with the relevant guidelines and regulations of the Institution.

### Cell culture: revival and expansion of bone marrow-mesenchymal stem cells (BM-MSC), adipose tissue-derived mesenchymal stem cells (AD-MSC) and dental pulp-derived mesenchymal stem cells (DP-MSC)

Cryopreserved BM-MSC, AD-MSC and DP-MSC (*N* = 05 each) were used for the study. They were revived, expanded and characterized as described by our group earlier^7^. Followed by characterization by flow cytometric enumeration and trilineage differentiation (data not shown), cells from 3rd to 5th passage were used for all the further experiments^7^. All the analytical experiments were performed in triplicates of each biological sample.

#### In vitro neuronal differentiation

For neuronal differentiation, induction medium containing neurobasal media (#10888-022, Gibco, USA), B27 supplement (Gibco, USA), EGF (#PHG0314, Gibco, USA), FGF2 (#10018B, Peprotech Asia) (10 ng/mL each) (PeproTech Asia) and 22-HC (2 µM) (#H5884, Sigma, USA), l-glutamine (Gibco, USA), PenStrep (Gibco, USA) was used. The induction protocol was carried out for 14 days with media change on every 3rd day. After the termination of the induction period at 14 days, the cells were used for further analytical experiments.

### Neurites’ length analysis

This was performed as per the already established protocol of the lab^7^. Briefly, induced hMSCs were examined for morphological changes under an inverted microscope. Images were captured and analysed using SI Viewer software (Tokyo, Japan) for the number and length of neurites, length of axon and area and diameter of the cell body. Respective uninduced hMSCs were used as experimental control^6^.

### Scanning electron microscopy (SEM)

Samples for SEM analysis were processed as per the established protocol of the lab^44,45^. Briefly, hMSCs were cultured and differentiated over cover slips. These samples on coverslips were collected and fixed with Karnovsky fixative (4% paraformaldehyde and 1% glutaraldehyde in 0.1 M phosphate buffer (pH 7.4)) for 6–8 h at 4 °C. Dried samples were mounted over aluminium stubs and sputter-coated with gold prior to imaging with EVO18 scanning electron microscope (Zeiss, Oberkochen, Germany) at 5 KVA in secondary electron imaging mode.

### Transmission electron microscopy (TEM)

Samples for TEM analysis were processed as per the established protocol of the lab^45^. After differentiation of hMSCs into neuronal cells, medium was removed and cells were given a gentle wash using PBS (pH 7.4), followed by fixation of cells by Karnovsky’s fixative (4% paraformaldehyde and 1% glutaraldehyde in 0.1 M phosphate buffer (pH 7.4)) for 6–8 h at 4 °C. After fixation, cells were washed gently with PBS. Cell numbers were such that they form a pellet of 100 µL upon centrifugation. Water from the cells was removed by treating them with a series of ascending concentrations of the dehydrating agent, ethanol. Ethanol was cleared by treating the cells with xylene. After this, samples were dehydrated in ascending grades of acetone and embedded in araldite CY212. Thin sections (70 nm) were cut with a glass knife and mounted onto nickel grids. They were contrasted with uranyl acetate and lead citrate and viewed under a transmission electron microscope (Tecnai, G 20 (FEI)).

### Transcriptional characterization of MSC induced into neuronal cells: quantitative reverse transcription-polymerase chain reaction (qRT-PCR)

After differentiation, total RNA from all the experimental groups was extracted by phenol–chloroform method as previously reported by our lab^7^. Single-strand cDNA synthesis was performed by using cDNA synthesis kit from ThermoFisher Scientific (#4368814, California, USA) according to the manufacturer’s protocol. Expression of Nestin, neurofilament (NF), microtubule-associated protein (MAP2) and tyrosine hydroxylase (TH) was studied in both induced and uninduced MSC. All these primers were obtained from Sigma (Missouri, USA) (data not shown).

qRT-PCR experiments were performed using Realplex real-time PCR detection system (Eppendorf, Germany), using SYBR green chemistry (#S4438, Kapa Biosystems, USA) as previously described^7^. qRT-PCR was done for Nestin, NF, MAP2, β III tubulin (Tuj1), TH and transcription factors, PitX3 and Ngn2. To study the genes related to dopamine transportation, expression of the dopamine transporter (DAT) gene was studied. Apart from these, genes related to various ion channels like Kv4.2 (potassium channel) and SCN5A (sodium channel) were also studied. Expression of nuclear receptors of 22-HC, i.e., LXRα and LXRβ were also studied. Primers of qRT-PCR grade were procured from Sigma (Missouri, USA).

The expression of the genes of interest was normalized to that of the housekeeping gene, glyceraldehyde-3-phosphate dehydrogenase (GAPDH). Melting curve was used to confirm the results and data were analysed using the graph pad prism software^7^.

### Immunocytochemistry

The assay was performed as previously described^7^. Briefly, fixed cells were incubated overnight at 4 °C with primary monoclonal antibodies against Nestin (1:400, #ab6320), MAP2 (1:250, #ab32454), TH (1:200, #ab112) and DAT (1:200, # ab92868) (Abcam, USA). After the induction period, hMSCs were washed five times with PBS and incubated with AF488 and AF594 conjugated secondary antibodies (1:500, Abcam, USA) for 1 h at room temperature (RT). Finally, after washing five times with PBS, cells were counterstained with 4′,6-diamidino-2-phenylindole (DAPI) (#10236276001, Sigma, USA) to visualize the cell nuclei. Cells were washed thrice with PBS to remove excess DAPI stain. Stained cells were examined using a fluorescence microscope equipped with a digital camera (Nikon Eclipse 80i, Japan).

### Intracellular staining for flow cytometry

hMSCs after induction with both the induction protocols, were labelled for Nestin (1:100), MAP2 (1:200), TH (1:100), synaptophysin (1:100, ab14692), DAT (1:100), GFAP (1:100, #ab4674), TPH2 (1:150, #ab111828), Ach (1:150, #ab2803) and S100 (1:100, #ab124805) as previously described^7^. Same antibodies were used for all the experiments. The dilutions used were titrated before performing final experiments. Briefly, the cells were incubated with primary antibodies for 1 h 20 min at 4 °C, followed by washing and incubation with secondary antibody labelled with fluorochrome-tagged secondary antibody (dilution of 1:400, abcam, USA) for 30 min at RT. The cells were then washed and suspended in PBS and acquired on BD LSR II flow cytometer (Becton Dickinson, USA) with a minimum of 10,000 events for each sample and analysed with FACs DIVA software (version 6.1.2). All the antibodies were procured from Abcam, USA.

### Immunoblotting

Immunoblotting for the expression of neuronal cell-specific proteins was performed with both induced and uninduced control cells, as previously described^7^. Briefly, after preparing whole cell lysates using RIPA buffer (#R0278, Sigma, USA), the protein quantification was done using bicinchoninic acid (BCA) assay method, according to the manufacturer’s protocol (#00-4333-57, Pierce, ThermoScientific, USA). Protein extracts (30 μg) were subjected to SDS–PAGE using 12% Tris/HCl sodium dodecyl sulphate (SDS) gels and transferred onto PVDF membranes (Membrane Technologies, India). After blocking the membranes with 3% BSA, they were incubated with primary antibody against β-actin (1:2500, #ab8227), MAP-2 (1:1500) and TH (1:1000, #sc-73151, Santa Cruz, USA) in 1% BSA-phosphate saline buffer (PBS) overnight at 4 °C. Post incubation, membranes were washed thrice in PBST and incubated with the appropriate horseradish peroxidase (HRP)-conjugated secondary antibody (1/4000) (Dako, USA) for 2 h at RT. Membranes were developed with chemiluminescence detection reagent (#34580, Pierce, USA) and acquired by using Gel Imager machine (Fluor Chem E, Cell Biosciences, Australia).

### Calcium ion imaging

Change in the concentration of calcium ions was studied by calcium ion imaging in hMSCs induced for 12 days in all study groups, as previously described^7^. Briefly, hMSCs upon induction, were stained with 10 µM of Fura red AM dye (#F3021, ThermoScientific, USA), mixed with the culture medium. The cells were incubated at 37 °C for 45 min. After incubation period, the cells were gently washed thrice with HBSS. Finally, the cells were activated using 50 mM KCl solution. Time lapse recording was made at 488 and 457 nm for 3 min. Baseline readings were obtained before adding KCl solution to the cells. The experiment was performed using Leica confocal microscope (model TCS SP8). The ratio of fluorescence at both the wavelengths was obtained and respective graphs were plotted. The experiment was performed on three samples each. The data was analysed using Leica LAS AF software.

### Establishment of PD Wistar rat model by creating unilateral 6-OHDA lesion in the midbrain

PD Wistar rat model was prepared as per the previously described protocol^46,47^. The male Wistar rats were anaesthetized with intra-peritoneal injections of ketamine (140 mg/kg body weight) and xylazine (30 mg/kg body weight). The rat was mounted on the stereotaxic machine and the operational skin area was shoved and disinfected with a povidone iodine solution (10% w/v, Johnson & Johnson, India) to locate bregma and lambda. The coordinates were set on the automatic mechanized stereotaxic machine for substantia nigra par compacta region of the brain (AP = −4.8 mm, PL = −2.2 mm and DV = −8.2 mm). Drilling was done with a movement of 30–50 microns. After the skull was drilled, the drilling stub was replaced with a 5 µL volume Hamilton syringe (Hamilton, USA), filled with 6-OHDA solution (Sigma, USA) (12 µg made in 0.2% ascorbic acid). The needle was taken in a controlled manner to 8.2 mm horizontally in the brain using the sterotaxic machine and 3 µL of 6-OHDA was released at the site in a rate-controlled manner. The needle was left there for 15 min to avoid retraction of 6-OHDA. After slowly removing the needle, the skin was sutured. To avoid oxidation of 6-OHDA, this whole process was performed in the dark. Postoperative care was given to the operated rats. Establishment of Parkinsonism in rats was confirmed by an apomorphine test^48^. All the animal experiments were randomized. The analytical experiments were performed in triplicates for each biological sample obtained.

### Behavioural assessment

#### Balance beam test

The beam test was performed to study the motor coordination between the forelimbs and hind limbs after creation of lesion using 6-OHDA. The beam set up was prepared according to the measures mentioned earlier^49^. The training protocol for the balance beam has been referred from the study by Brooks et al. (2009)^50^. Briefly, all rats were tested on the balance beam apparatus for 3 days consecutively. The rats were habituated for the first 2 days by training them to traverse the beam to and forth from the goal box to the starting point. On the third day, the rats were videotaped for three consecutive runs and the time taken to traverse the whole beam or for first foot drop was calculated while moving.

### Fluorescence in situ hybridization (FISH) analysis

FISH analysis was performed with the specific probes for human chromosome X as per the protocol described earlier^51^. Briefly, cells over the slides were cleared with xylene for 10 min, and twice re-hydrated in several washes with 100%, 85% and 70% ethanol for 5 min each. Then slides were rinsed with distilled water for 5 min at RT. For enzymatic digestion, hydrochloric acid 0.2 M was used for 10 min, and slides were continuously treated with Proteinase K 10 mg/mL for 10 min in a bath at 37 °C. Slides were rinsed with saline–sodium citrate buffer for 5 min and fixed with 1% paraformaldehyde buffer for 10 min before being rinsed again with citrate buffer for 5 min and air dried. Next, slides were dehydrated ethanol prior to hybridization with the probes. The mixture of probes was added to the slides, covered with coverslips and incubated in a humidified chamber at 80 °C for 5 min for denaturation and at 37 °C for 72 h for hybridization. Afterwards, slides were washed with the buffer in a bath at 80 °C for 2 min and then at RT. Finally, nuclei were counter-stained with diamidino-2-phenylindole dihydrochloride (DAPI). All the experimental controls were taken under this experiment.

### Immuno-histochemical analyses

Immunohistochemical staining for MAP2 and TH markers of mature neurons and DAergic neurons, respectively, was performed using antibodies specific for humans using previously published protocol from the lab^44^. Prior to immunostaining, rat brain tissue sections were incubated at 95 °C for 30 min in target antigen retrieval buffer (10 mM Tri-sodium citrate containing 0.05% Tween 20, pH 6.0) for epitope recovery. Tissue specimens were incubated overnight at 4 °C with primary antibodies mouse antihuman MAP2 (1:50), and rabbit antihuman TH (1:50). The sections were further incubated with secondary antibodies: respective HRP-linked secondary antibodies (1:200) for 1 h 20 min at RT. Nuclear staining was performed using DAPI (5 mg/mL; 1:4000) for 3 min at RT. The specimens were further observed under microscope (Olympus,IX71, Tokyo, Japan).

### Data interpretation and statistical analysis

Means ± SD of independent experiments were analysed by the Student’s *t*-test, Tukey’s test, one way and two way ANOVA test (as per the requirement of data analysis). *p* < 0.05 was considered as statistically significant. Analysis of data was done by using GraphPad Prism 5.00 software (San Diego, CA, USA).
